# A systematic review and meta-analysis of the prevalence and global distribution of middle mesial canals in mandibular molars identified by CBCT

**DOI:** 10.1007/s00784-024-05660-z

**Published:** 2024-05-14

**Authors:** Mahmood Reza Kalantar Motamedi, Mohammad Hosein Amirzade-Iranaq, William N. Ha

**Affiliations:** 1 Dental Research Center, Dental Research Institute, School of Dentistry, Isfahan University of Medical Sciences, Isfahan, Iran; 2 Department of Research & Development, Farinroshaan Medical & Health Co. Ltd., Tehran, Iran; 3 Faculty of Medicine and Health, Sydney Dental School, The University of Sydney, Surry Hills NSW, Sydney, 2010 Australia

**Keywords:** Middle mesial canal, Root canal, Anatomy, Endodontics, Systematic review

## Abstract

**Objectives:**

This systematic review and meta-analysis aimed to evaluate the prevalence of middle mesial canal (MMC) in permanent mandibular molars of different populations and regions based on cone-beam computed tomography (CBCT) studies.

**Materials and methods:**

PubMed, Scopus, Embase, Web of Science, and Open-Grey were searched up to October 2023 according to specific keywords. A hand search was conducted on the references of the included studies and articles from three peer-reviewed journals in endodontics. The main variable of interest was the prevalence of MMC. Additional data such as the total number of included cases, age and country of the population, CBCT device information, voxel size, and field of view details were also extracted. Extracted data were analyzed qualitatively with the JBI quality assessment checklist and quantitatively with STATA software.

**Results:**

Of 32,793 studied teeth, the cumulative prevalence of MMC in both mandibular 1^st^ and 2^nd^ molars was 3.11% (95% CI: 2.00-4.44%). The subgroup analysis reveals a prevalence of 4.15% (95% CI: 2.69-5.89%) for mandibular 1^st^ molars and 1.2% (95% CI: 0.2-2.83%) for mandibular 2^nd^ molars. The highest prevalence of MMC in 1^st^ molar was attributed to South Asia (11.24%) and Africa (6.61%).

**Conclusions:**

The prevalence of MMC varies among regions. Clinicians should be aware of the potential prevalence of MMC, particularly in mandibular first molars, as a missed MMC could result in endodontic failure.

**Clinical relevance:**

The presence of MMCs varies in different geographic regions (0% to 29.7%). Clinicians should always look for MMC when doing an endodontic treatment on mandibular molars, as the presence of this canal is not uncommon. We suggest searching for this canal as if searching for the second mesiobuccal canal of maxillary 1st molars.

**Supplementary Information:**

The online version contains supplementary material available at 10.1007/s00784-024-05660-z.

## Introduction

The inability to identify and debride all infected root canals can contribute to failure in root canal treatment [1, 2]. The complexities of anatomy can limit the ability of clinicians to see or find root canals and hence reduce their ability to disinfect and adequately obturate the canals [2].

Mandibular 1^st^ and 2^nd^ molars have been commonly described as having two roots, one mesial and one distal [3, 4], with the mesial root having a mesiobuccal (MB) and a mesiolingual canal (ML), while the distal root has a single canal [5]. A variation is an additional canal originating between the MB and the ML canal, referred to as the middle mesial canal (MMC) [6]. Due to the poor accessibility of the MMC, this canal might act as a reservoir for residual infected pulp tissue and bacteria, leading to persistent apical periodontitis [2, 7].

A systematic review assessed MMC’s prevalence, finding that MMCs ranged between 0.26-53.8% [8]. This wide range was related to the included studies that varied in methodology. The methods of the studies included the clearing technique, conventional radiography, scanning electron microscope, dental operating microscope (DOM), cone-beam computed tomography (CBCT), and micro-CT. Moreover, their search keywords were somehow limited and were not comprehensive.

Micro-CT studies are accurate and can reveal details of the root canal anatomy. However, the sample size of such studies is often limited due to the expenses of such studies [9]. Moreover, they can only be performed on extracted teeth that are not representative of healthy teeth because extracted teeth may have been associated with endodontic or periodontal disease [10]. Furthermore, these studies are primarily performed on a pool of extracted teeth; therefore, they cannot differentiate between the extracted 1^st^, 2^nd^, and 3^rd^ molars.

Clinical in vivo studies with an operating microscope can explore negotiable MMCs in non-extracted teeth [11, 12]. However, it may be difficult to distinguish a true MMC from an isthmus [11]; therefore, a higher incidence of MMC may be found in such studies.

CBCT is as accurate as micro-CT for identifying canals and is clinically realistic for what can be identified when treating patients [9]. CBCT has the advantages of being *in vivo* and non-invasive and allows for expanding the sample size compared to microscopic analysis or other laboratory studies using extracted teeth. CBCT is an appropriate in vivo tool for evaluating anatomic variations and has been widely used to study large populations [13, 14]. In addition, detailed epidemiological data such as gender, race, and ethnicity can also be obtained from CBCT studies.

Recently, a meta-analysis was performed on the global prevalence of MMC, focusing primarily on CBCT studies [15]. They reported the pooled prevalence rates on 4 continents (Asia, Europe, America, and Africa). However, categorizing into only 4 continents and reporting the prevalence based on such vast continents seems biased. For example, there are different races in East Asia compared to West Asia or North America compared to South America. Moreover, after their study, some new articles in new regions with considerable sample sizes were published; the most prominent one is a very well-designed multinational study with a sample size of 12,608 teeth [16].

Thus, this study aimed to perform a systematic review of the literature and meta-analysis to evaluate the prevalence of MMC in permanent mandibular molars of different populations based on CBCT studies.

## Material and methods

This systematic review was reported following the Preferred Reporting Items for Systematic Reviews and Meta-Analyses statement (PRISMA) [17]. The protocol of this review was registered in PROSPERO under the code number CRD42022375405.

### Eligibility criteria

Studies using CBCT to evaluate MMC prevalence in human permanent mandibular molars, except third molars, with completely developed apices, and without previous endodontic treatment, were included. Moreover, only studies using voxel sizes equal to or below 200 μm were included [4]. *In vitro* studies were excluded. No language limitation was applied. Review articles, case reports, surveys, comment letters, book chapters, and conference abstracts were excluded.

### Information sources

PubMed, Scopus, Embase, and Web of Science were searched with specific search terms up to the 1^st^ of October 2023. Two separate reviewers performed the screening process for studies evaluating the prevalence and morphology of MMC in permanent human mandibular molars using CBCT. In addition, a gray literature search was conducted in Open-Grey (opengrey.org) and Google Scholar. Finally, three peer-reviewed endodontic journals (International Endodontic Journal, Journal of Endodontics, and Australian Endodontic Journal) and the references of the included studies were manually searched to identify relevant literature. Possible relevant articles were investigated during the screening process by considering references and citations from included studies and relevant published reviews. When necessary, authors were contacted to request unavailable data or analyses.

### Search strategy

The search syntax was developed with the direct supervision of two expert endodontists. First, keywords such as “mid mesial canal,” “root canal morphology,” “molar,” “mandibular molar,” “posterior teeth,” “cone-beam computed tomography,” “CBCT,” and “prevalence” were used to construct the main body of the syntax. Synonyms, Boolean operators, field tags, and MeSH terms (if applicable) were added in the following steps to finalize the syntax. The search terms and filters used to search PubMed (MEDLINE and PMC) to identify relevant studies are shown in Supplementary Table S1. (By searching other databases with similar syntax and adapting them to match the operators of each database, we have maximized the effectiveness of our search.)

### Studies selection process

Titles and abstracts of the articles were screened according to reviewers’ predefined inclusion and exclusion criteria, and “relevant” articles were subjected to full-text screening by two separate reviewers. Discussion with a senior endodontist resolved any disagreements between the two reviewers.

### Data collection and data items

Data extraction was summarized using Microsoft Excel (Microsoft, Redmond, WA), and two independent reviewers collected the data independently. Afterward, disagreements were resolved through discussion among the reviewers. The data extraction form contained the following details: study title, author/year, sample size, tooth type, age, gender, and geographic location. In addition, CBCT parameters such as machine brand and voxel size used in that particular study were also tabulated.

To extract the number of MMCs, we considered MMC as

1. an extra canal between mesiobuccal (MB) and mesiolingual (ML) canals [10] or
2. any specific modifications of root canal classifications with three or more root canals in a single root [18]. For example, Vertucci’s type VIII [5], additional modifications of Vertucci’s canal types introduced by Gulabivala et al. (except type X) [19], all types of Pomeranz et al. classification [6], Sert and Bayirli classification types VIII to XXIII (except type XIX) [20], and Kartal’s type VI and VII [21].

Variables were extracted into two main categories. The first category included demographic characteristics such as the author’s name, study year, and location. The second category comprised the total number of studied teeth, the prevalence of MMC, and tooth type (mandibular 1^st^ or 2^nd^ molar).

### Risk of bias assessment

Risk of bias assessment of the included articles was performed using the Joanna Briggs Institute (JBI) critical appraisal tool for prevalence data [22]. Two evaluators independently assessed eligible studies and scored each JBI question as “yes”, “no”, or “unclear”. Discrepancies in the assessment were discussed with senior team members until a consensus was reached. The final score of each article subjected to the JBI appraisal was calculated based on the percentage of positive answers (“yes”). In addition, it was classified as having a “high” risk of bias when the score was <50%, a “moderate” risk of bias if the score ranged from 50% to <70%, and a “low” risk of bias if the score was >70%.

### Synthesis methods

The DerSimonian-Laird bivariate random-effects model analysis was performed with STATA software version 16 (StataCorp, College Station, TX) with the “metaprop” package written by VN Nyaga [23]. In addition, the Freeman-Tukey Double Arcsine Transformation method was used to stabilize the variances by studying confidence intervals for analyzing studies involving a small sample size and proportions value that were too high (towards 1) or low (towards 0).

## Results

**Fig. 1:**
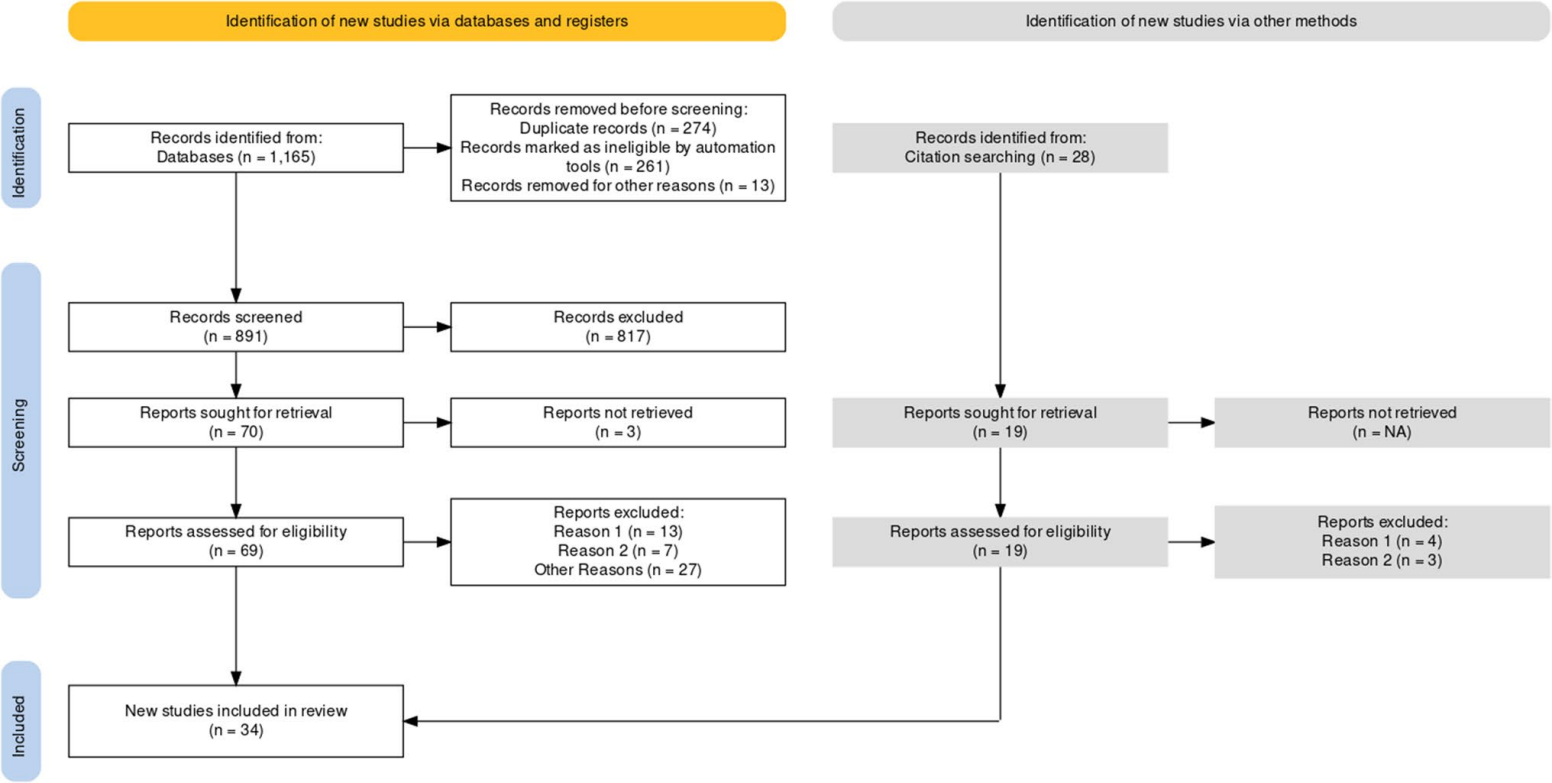
PRISMA flow diagram regarding the process of identification of studies

A total of 1165 articles were obtained from the electronic database search, gray literature, and hand searching. After applying the eligibility criteria and eliminating duplicates, 88 articles were selected for full-text assessment. Following the reading of the full-texts, 54 studies were excluded. Thus, 34 studies that fulfilled the eligibility criteria were included for quality assessment and quantitative synthesis (Fig. 1 & Supplementary Table S2).

### Included studies characteristics

A total of 34 studies reporting the prevalence of MMC in mandibular molars were included in the meta-analysis from 27 countries and seven geographical regions reporting data from 32,793 studied teeth (Tables 1 and 2).

**Table 1:** Summary of extracted data from included studies on the prevalence of middle mesial canal in mandibular 1^st^ molars

First author, year	Country	CBCT device	Voxel size (μm)	FOV (cm)	Age (years)	Total No. of teeth	Overall MMC prevalence (%)
Almedia, 2023 [24]	Brazil	Eagle 3D unit (Dabi Atlante, Ribeirão Preto, Brazil)	200	8 × 12	18-75 (Mean age: 28.2±11.7)	242	0 (0%)
Alroomy, 2022 [25]	Saudi Arabia	Orthophos SL 3D (Dentsply Sirona, Bensheim, Germany)	160	5 × 5	ND	200	10 (5%)
Arayasantiparb, 2017 [26]	Thailand	3D Accuitomo XYZ Slice View Tomograph (J. Morita, Kyoto, Japan)	125	6 × 6	11-68	518	2 (0.38%)
Bhatti, 2022 [27]	Pakistan	Planmeca ProMax 3D (Planmeca, Helsinki, Finland)	200	4.2 × 4.2	ND	298	23 (7.7%)
Caputo, 2016 [28]	Brazil	Gendex CB500 (Gendex Dental Systems, Des Plaines, IL)	200	8 × 14	19-81 (Mean age: 48.9)	342	3 (0.8%)
Drouri, 2022 [29]	Morocco	NewTom VGi (NewTom, Verona, Italy)	125	8 × 8	ND	396	1 (0.3%)
Hasheminia, 2021 [30]	Iran	Scanora 3D (Soredex, Tuusula, Finland)	133 and 200	6 × 6	ND	768	24 (3.13%)
Hatipoglu, 2023 [16]	Croatia	Cranex 3D (Soredex)	200	5 × 10	Mean age: 32.87±13.74	326	4 (1.23%)
	Egypt	Planmeca Promax 3D	150	8 × 8		1006	12 (1.2%)
	Germany	Planmeca Promax 3D	160	8 × 8		496	77 (15.52%)
	India	CS 9600 (Carestream Dental, Atlanta, Ga)	150	8 × 9		982	97 (9.9%)
	Jordan	CS 8100SC 3D (Carestream Dental)	150	8 × 9		1004	18 (1.8%)
	Kazakhstan	Orthophos XG 3D (Sirona, Salzburg, Austria)	100	8 × 8		998	54 (5.41%)
	Libya	CS 8100 (Carestream Dental)	150	8 × 9		1006	233 (23.16%)
	Malaysia	NewTom Giano HR (QR SRL, Verona, Italy)	150	13 × 8		1038	17 (1.63%)
	Pakistan	Planmeca Promax 3D	200	13 × 19		320	23 (7.18%)
	Poland	Cranex 3Dx (Soredex)	200	6 × 8		1080	12 (1.1%)
	Portugal	i-CAT (i-CAT, Hatfield, England)	200	10 × 8		1216	46 (3.8%)
	Saudi Arabia	Galileos Comfort (Sirona, Beinshiem, Germany)	160	15 × 15		508	65 (12.8%)
	South Africa	Planmeca Promax 3D	200	10 × 9		786	102 (13%)
	Turkey	3D Accuitomo 170 (J. Morita)	200	10 × 10		1336	106 (8%)
	Yemen	Pax–I 3D Green (Vatech, Hwaseong, Korea)	170	12 × 9		506	12 (2.4%)
Hosseini, 2020 [31]	Iran	NewTom 5G (NewTom)	75	8 × 12	ND	200	18 (9%)
Hu, 2019 [32]	China	DCTPRO (Vatech, Yongin-Si, Korea)	160	16 × 7	12-70 (Mean age: 38.4)	823	89 (10.9%)
Inaty, 2020 [18]	Lebanon	NewTom VGi; Kodak 9500 3D (Carestream Health, Marne-la-Vallée, France)	200	7 × 12	Mean age: 38.08±15.1	242	32 (13.2%)
Iqbal, 2022 [33]	India	CS 9300 (Carestream Dental)	180	5 × 10	15-40 (Mean age: 28 for males, 26 for females)	212	63 (29.7%)
Kantilieraki, 2019 [34]	Greece	NewTom VGi Evo (NewTom); Scanora 3D	100	ND	18-65 (Mean age: 37)	477	1 (0.21%)
Kim, 2013 [35]	South Korea	Dinnova (Willmed, Gwangmyeong, Korea)	167	10 × 10	13-69 (Mean age: 28.8)	1939	5 (0.26%)
Martins, 2018 [36]	Portugal	Planmeca Promax 3D	200	Full arch	Mean age: 51	463	25 (5.4%)
	China	Kodak 9500 (Carestream Dental)	200	Full arch	Mean age: 28	220	0 (0%)
Pérez-Heredia, 2017 [37]	Spain	CS 9300 3D (Carestream Dental)	180	10 × 10	18-62 (Mean age: 36.8)	119	10 (8.4%)
Popović, 2020 [38]	Serbia	Orthophos XG 3D	160	ND	ND	118	0 (0%)
Qiao, 2020 [39]	China	3D Accuitomo	125	6 × 6	ND	1174	21 (1.79%)
Rehman, 2020 [40]	Pakistan	Planmeca Promax 3D Max (Planmeca)	200	ND	Mean age: 31	94	6 (6.4%)
Senan, 2020 [41]	Yemen	Pax-Flex3D (Vatech, Hwaseong, Korea)	120	5 × 5	18-40	500	15 (3%)
Shakeri, 2019 [42]	Iran	Cranex 3D	130	6 × 8	ND	207	7 (3.4%)
Silva, 2103 [43]	Brazil	i-CAT	200	8 × 8	ND	230	0 (0%)
Tahmasbi, 2017 [10]	United States of America	CS 9000 3D (Carestream Health, Rochester, NY)	76	5 × 3.7	Mean age: 45	57	15 (26.32%)
Wang, 2010 [44]	China	3D Accuitomo MCT-1 (J. Morita)	125	ND	12-75	554	13 (2.35%)
Xu, 2020 [45]	China	NewTom VGi	125	8 × 8	9-81 (Mean age: 41.24)	357	11 (3.1%)
Yang, 2020 [46]	China	Planmeca Romexis 3D (Planmeca)	200	8 × 8	Mean age: 37.9±1.76	1750	158 (9.03%)
Zhang, 2011 [47]	China	3D Accuitomo	125	4 × 4 or 6 × 6	18-57 (Mean age: 37)	232	0 (0%)
Zhang, 2015 [48]	China	Galileos (Sirona)	125	ND	ND	909	0 (0%)

**Table 2:** Summary of extracted data from included studies on the prevalence of middle mesial canal in mandibular 2^nd^ molars

Author, year	Country	CBCT device	Voxel Size (μm)	FOV (cm)	Age (years)	Total No. of teeth	Overall MMC prevalence (%)
Almedia, 2023 [24]	Brazil	Eagle 3D unit (Dabi Atlante; Ribeirão Preto, Brazil)	200	8 × 12	18-75 (Mean age 28.2±11.7)	242	3 (1.24%)
Arayasantiparb, 2017 [26]	Thailand	3D Accuitomo XYZ Slice View Tomograph (J. Morita, Kyoto, Japan)	125	6 × 6	11-68	385	0 (0%)
Donyavi, 2019 [49]	Iran	Cranex 3D (Soredex, Tusuula, Finland)	200	6 × 8	Mean age: 39.06 ±12.72	406	0 (0%)
Gomez, 2021 [50]	Venezuela	Kodak 9000 3D (Carestream Dental, Atlanta, GA)	76	ND	ND	153	0 (0%)
Inaty, 2020 [18]	Lebanon	NewTom VGi (NewTom, Verona, Italy); Kodak 9500 3D (Carestream Health, Marne-la-Vallée, France)	200	7 × 12	Mean age: 38.08±15.1	263	42 (16%)
Iqbal, 2022 [33]	India	CS 9300 (Carestream Dental, Atlanta, Ga)	180	5 × 10	15-40 (Mean age: 28 for males, 26 for females)	288	46 (16%)
Kantilieraki, 2019 [34]	Greece	NewTom VGi Evo (NewTom); Scanora 3D (Soredex)	100	ND	18-65 (Mean age: 37)	460	0 (0%)
Kim, 2016 [51]	South Korea	Dinnova (Willmed, Gwangmyeong, Korea)	167	10 × 10	13-75 (Mean age: 28.7)	1148	0 (0%)
Martins, 2018 [36]	Portugal	Planmeca Promax (Planmeca, Helsinki, Finland)	200	Full arch	Mean age: 51	589	13 (2.21%)
	China	Kodak 9500 (Carestream Dental)	200	Full arch	Mean age: 28	131	0 (0%)
Pawar, 2017 [52]	India	Planmeca ProMax 3D Mid (Planmeca)	100	5 × 4 to 16 × 16	15-60 (Mean age: 26.8)	983	0 (0%)
Pérez-Heredia, 2017 [37]	Spain	CS 9300 3D (Carestream Dental)	180	10 × 10	18-62 (Mean age: 36.8)	101	3 (3%)
Popović, 2020 [38]	Serbia	Orthophos XG 3D (Sirona, Salzburg, Austria)	160	ND	ND	162	0 (0%)
Rehman, 2020 [40]	Pakistan	Planmeca Promax 3D Max (Planmeca)	200	ND	Mean age: 31	95	3 (3.16%)
Saber, 2023 [53]	Egypt	Promax 3D Classic (Planmeca)	75	5 × 5	15-65	292	0 (0%)
Senan, 2021 [54]	Yemen	Pax-Flex3D (Vatech, Hwaseong, Korea)	120	5 × 5	18-40	448	11 (2.45%)
Shakeri, 2019 [42]	Iran	Cranex 3D	130	6 × 8	ND	235	7 (3%)
Silva, 2103 [43]	Brazil	i-CAT (i-CAT, Hatfield, England)	200	8 × 8	ND	218	0 (0%)
Tahmasbi, 2017 [10]	USA	CS 9000 3D (Carestream Health, Rochester, NY)	76	5 × 3.7	Mean age: 45	65	5 (7.7%)
Zhang, 2011 [47]	China	3D Accuitomo	125	4 × 4 or 6 × 6	18-57 (Mean age: 37)	122	0 (0%)

### Prevalence of MMC in mandibular 1^st^ and 2^nd^ molars

**Fig. 2:**
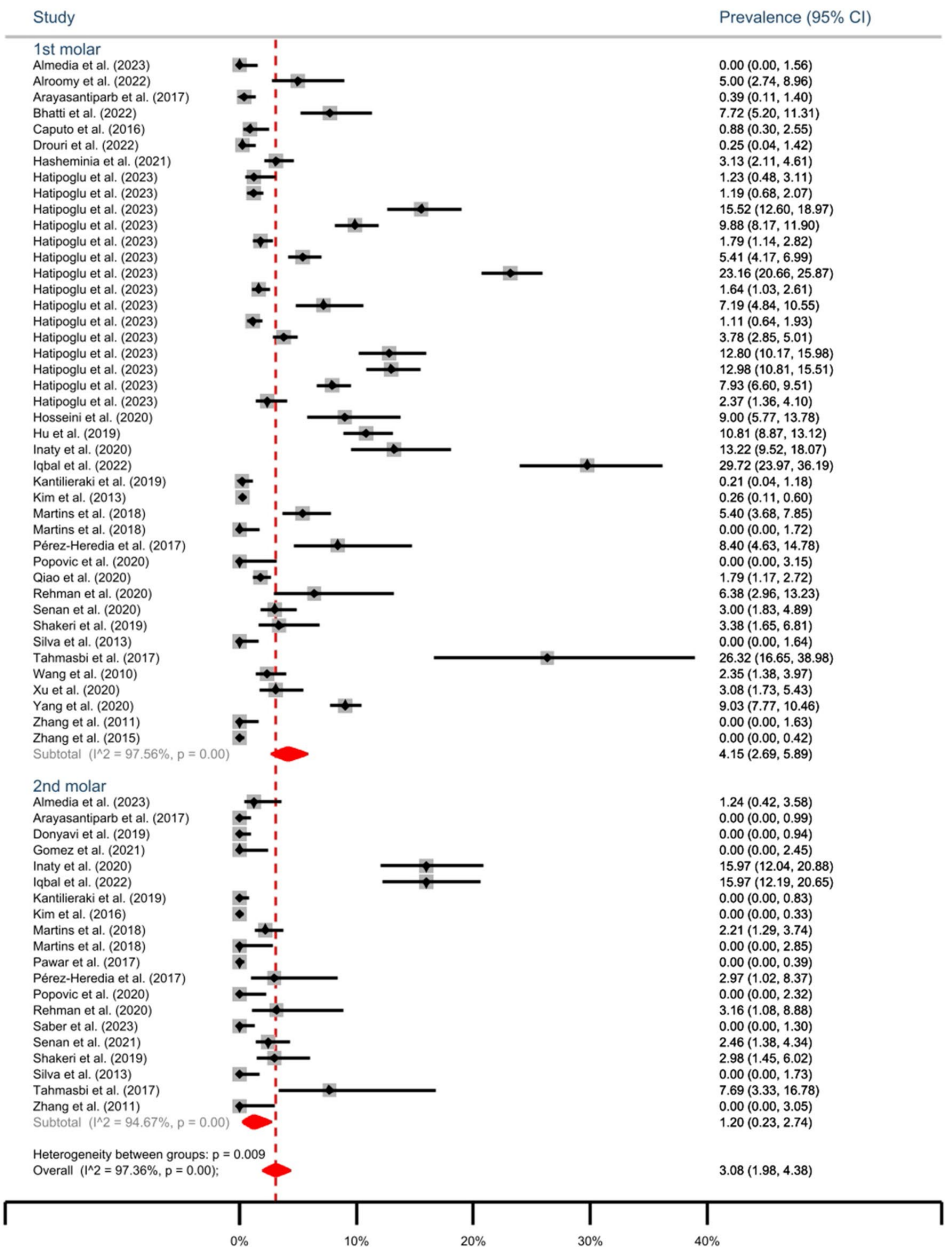
Forest plot of meta-analysis of the prevalence of middle mesial canal in mandibular 1^st^ and 2^nd^ molars

**Fig. 3:**
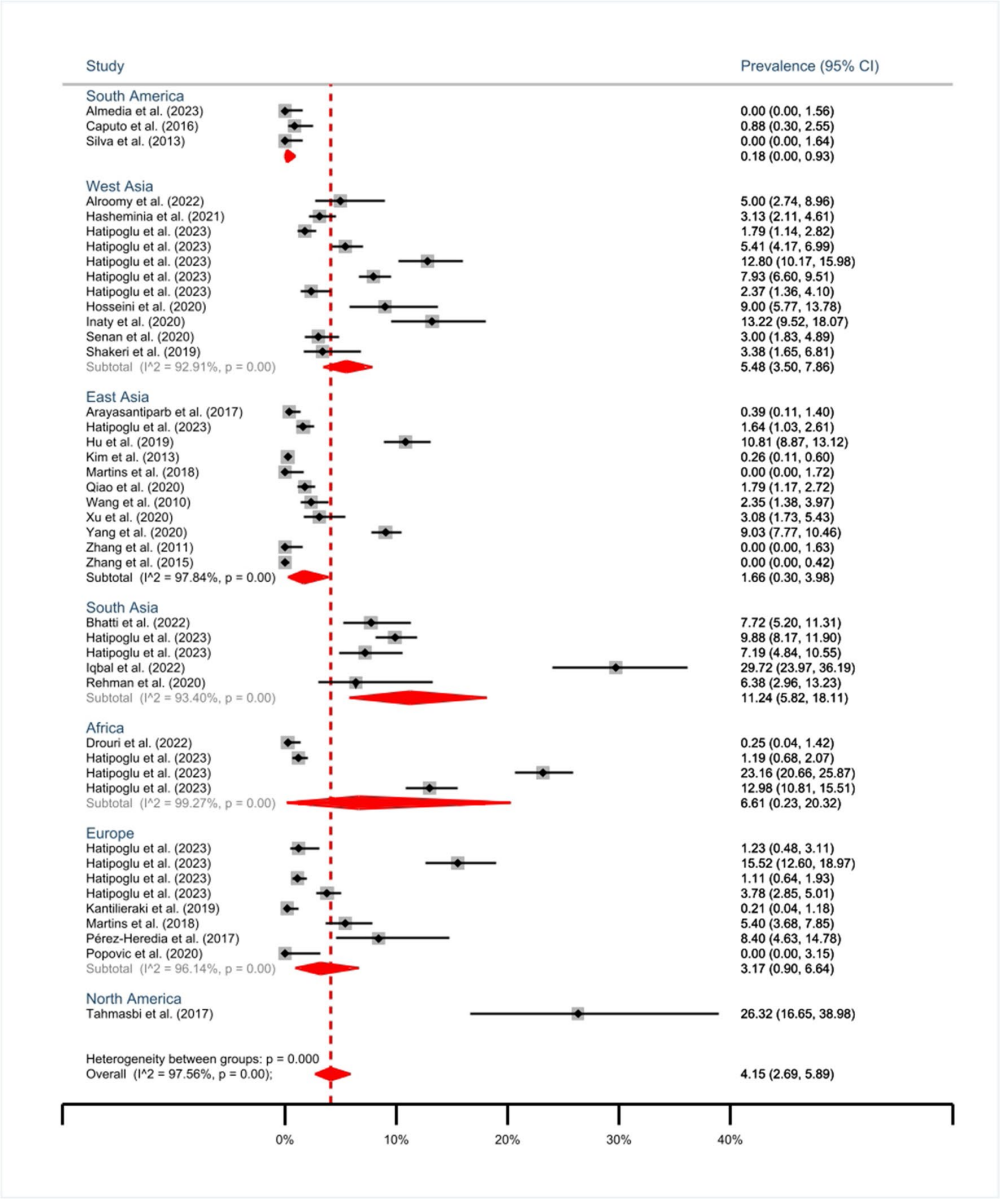
Forest-plot of subgroup meta-analysis of the prevalence of middle mesial canal in mandibular 1^st^ molars by geographical regions

The cumulative prevalence of MMC in both mandibular 1^st^ and 2^nd^ molars is 3.11% (95% CI: 2.00-4.44%). The subgroup analysis reveals a prevalence of 4.15% (95% CI: 2.69-5.89%) for 1^st^ molars and 1.2% (95% CI: 0.2-2.83%) for 2^nd^ molars (Fig. 2). The highest prevalence of MMC in mandibular 1^st^ molars was reported by Iqbal et al. as 29.72% [33] (Fig. 3).

**Fig. 4:**
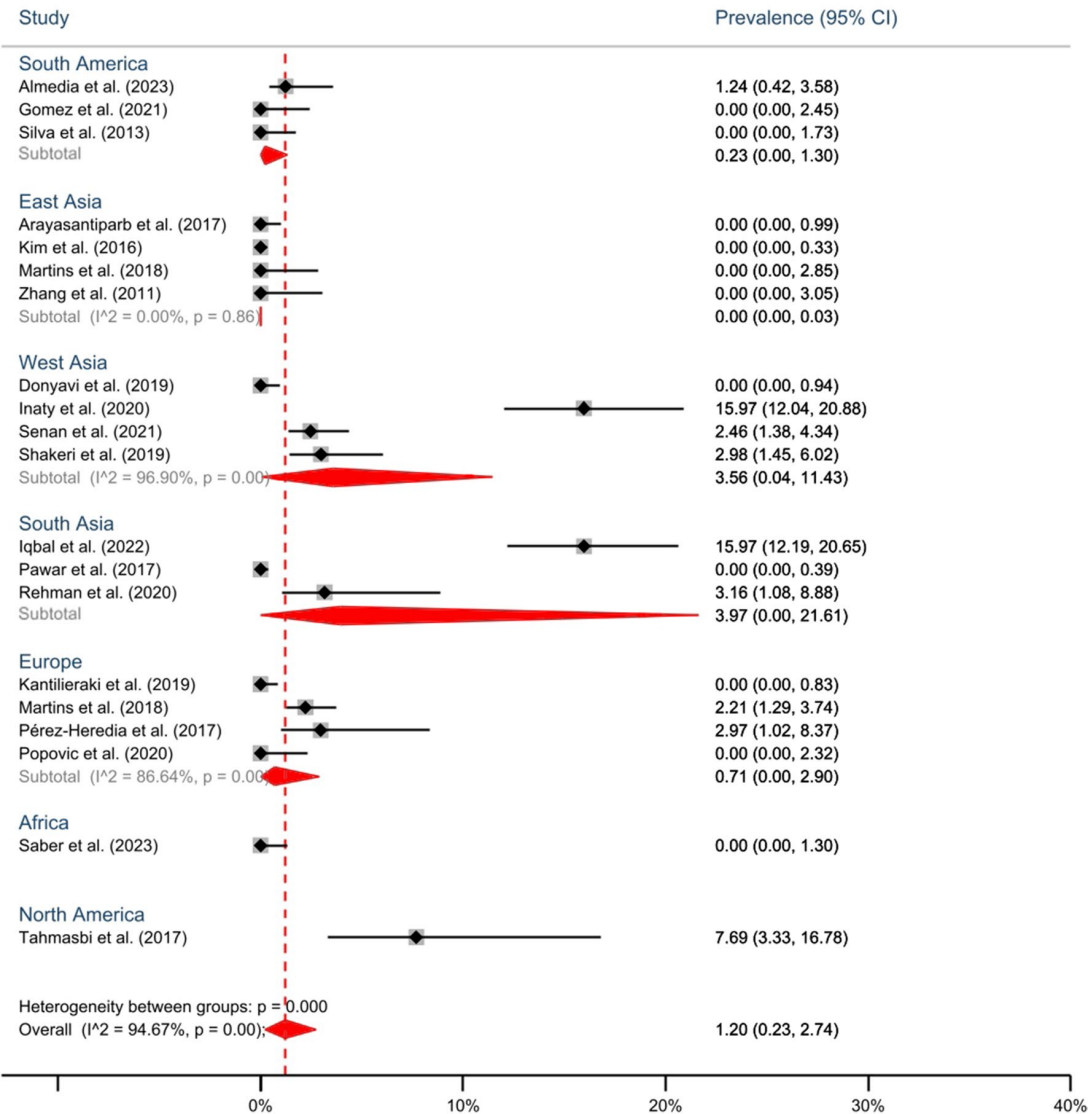
Forest-plot of subgroup meta-analysis of the prevalence of middle mesial canal in mandibular 2^nd^ molars by geographical regions

In mandibular 2^nd^ molars, the highest reported prevalence of MMC was in the studies of Inaty et al. [18] and Iqbal et al. [33], which was 16%. However, most studies reported no MMC in mandibular 2^nd^ molars (Fig. 4).

### Prevalence of MMC in mandibular molars by geographical region

The included studies reported a prevalence of MMC in mandibular 1^st^ and 2^nd^ molars were categorized into seven geographical regions as follows:

a) South Asia: India and Pakistan; b) West Asia: Iran, Saudi Arabia, Jordan, Kazakhstan, Turkey, Yemen, and Lebanon; c) East Asia: China, Korea, Thailand, Malaysia; d) Africa: Morocco, Egypt, Libya, South Africa, Egypt; e) Europe: Croatia, Germany, Poland, Portugal, Greece, Portugal, Spain, Serbia, Greece, Portugal, Spain, and Serbia; f) North America: USA; and g) South America: Brazil and Venezuela.

**Fig. 5:**
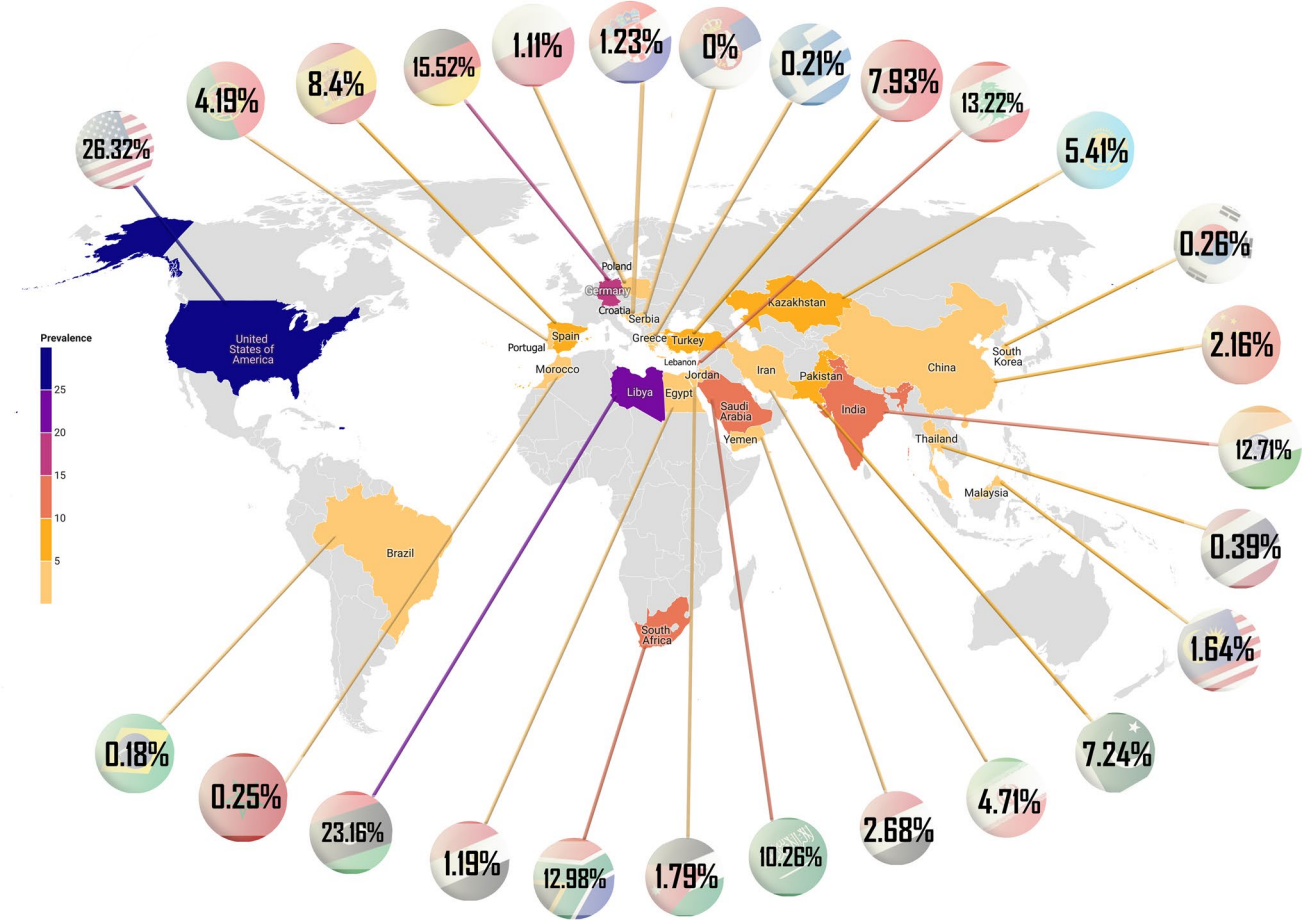
Prevalence of MMC in mandibular 1^st^ molars by different countries

According to the subgroup meta-analysis, the highest prevalence of MMC in mandibular 1^st^ molar is attributed to North America with 26.32% (95% CI: 13-22%) [10]. However, this result is from a single study. South Asia followed with 11.24% (95% CI: 5.82-18.11%), and Africa with 6.61% (95% CI: 0.23-20.32%), demonstrating the highest prevalence of MMC in mandibular 1^st^ molars than other regions (Fig. 3). Also, the details of the prevalence of MMC in the mandibular 2^nd^ molar are shown in Fig. 4. The prevalence of MMC in mandibular 1^st^ molars in different countries is depicted in a world map (Fig. 5).

### Studies quality assessment

A risk-of-bias assessment is provided in detail for each included study in Supplementary Figure S1. Low bias levels were attributed to 94.1% of studies, and moderate bias levels were attributed to 5.9% of studies. In addition, an overall quality assessment is provided in Supplementary Figure S2.

## Discussion

In the present study, the worldwide prevalence of MMC in the mandibular 1^st^ molar was 4.15%. However, the presence of MMC varies in different geographical regions and ranges from 0 to 29.7%. Except for the prevalence of MMC in the United States of America, which was based on only a single study (26%), the highest prevalence of MMC in the mandibular 1^st^ molar was found in South Asia (11.24%), followed by Africa (6.61%).

According to the results of the present study, a cumulative prevalence of 1.2% was found for the occurrence of MMC in the mandibular 2^nd^ molars. The highest prevalence of MMC in the mandibular 2^nd^ molar was found in the South Asia (3.97%) and the West Asia (3.56%).

In the present study, for the first time, the cumulative prevalence of MMC is reported by different countries (Fig. 5). Moreover, the prevalence of MMC is reported separately in different regions and continents of the world. Before our study, a systematic review assessed the prevalence of MMC [8]. However, their study had some limitations; for instance, the studies varied in methodology, and voxel sizes of more than 200 μm were included, which is unsuitable for detecting root canal anatomy [55]. Moreover, there was no mention of whether the studies excluded endodontically treated teeth [8]. These differences might depict why the prevalence range of MMCs varied in their study (0.26-53.8%) compared to ours (0-29.7%). Recently, Al-Maswary et al. published a meta-analysis on the global prevalence of MMC focused primarily on CBCT studies [15]. They reported the global prevalence of MMC to be 4.4% in the 1^st^ molar and 1.3% in the 2^nd^ molar, which was in accordance with the results of our study. However, they reported the pooled prevalence rates based on only 4 continents (Asia, Europe, America, and Africa). The racial discrepancy cannot be applied in such a classification based on the vast extent of the continents. For instance, there are different races in East Asia compared to West Asia or North America compared to South America. Therefore, in our study, the regions were classified more precisely including more number of regions but with less extent, including South Asia, West Asia, East Asia, Africa, Europe, North America, and South America. By doing this, the racial bias can be controlled. Compared to the study of Al-Maswary et al., we reported the pooled prevalence rates based on countries wherever possible; therefore, clinicians can find the prevalence of MMC in their country (Fig. 5). More importantly, based on the studies that were published after the study of Al-Maswary, we were able to include another 11,820 samples from new countries, including Saudi Arabia, Egypt, Germany, Croatia, Jordan, Kazakhstan, Libya, Malaysia, Poland, and Turkey.

A well-designed multinational cross-sectional study on the prevalence of MMC with a sample size of 12,608 reported a range of 1-23% [16]. In their study, the prevalence of MMC was 7% for the mandibular 1^st^ molar, which is more than the results of our study. The greater prevalence can be because their study does not include countries from East Asia, North America, and South America [16].

One of the limitations of our study is that regional prevalence cannot necessarily imply ethnic diversities [4]. A geographic region can have multiple ethnic origins, as many countries are a mix of ethnicities. For example, in the study by Pan et al. performed in Malaysia, the majority were Chinese (92.3%), followed by Indians (4.3%), Malays (2.4%), and other races (1.0%) [56]. Furthermore, the root canal anatomy of teeth may vary with sex and age [4]. In our study, the gender and age of the studied population could not be analyzed due to variations in measurements and frequently missed data. However, in general, the studies that focused only on the prevalence of the MMC canal did not find any difference in gender [10, 16, 30, 57].

As mentioned above, no meta-analysis was performed on age and its relation with the prevalence of MMC because of the insufficient data and heterogeneity of the most included studies regarding age. However, some studies reported that the prevalence of identifiable MMC decreases as age increases [8, 46]. Therefore, the prevalence of identifiable MMC could be related to secondary dentin deposition [58]. In contrast, Tahmasbi et al. [10] found the highest prevalence of MMC in the 41-60 age group, and Srivastava et al. [57] found the highest prevalence of MMC in the 31-50 age group. The contrast in findings could also be due to the formation of dentin within the isthmus between the MB and the ML canal, where a previously joined canal becomes separated by dentin and thus creates another canal.

It is also essential to differentiate and clarify the meaning of the isthmus and MMC. Bansal et al. defined an isthmus as a narrow connection between two root canals containing pulp tissue [8]. Pomeranz et al. described MMC as a fin, confluent, or independent canal between the MB and ML canals [6]. However, this definition cannot distinguish between an isthmus and an actual canal [10]. Some have defined a “true MMC” as a clear, round cross-section in the radiographic image between the MB and ML canals, which can be with an isthmus [10, 45]. A systematic review discussed that the inconsistent definition of MMC might be a reason for the wide range of MMC occurrences [8].

Another reason for the diversity of the data in the literature might be the different detection methods to identify MMC. For example, clinical *in vivo* studies with a DOM can explore negotiable MMCs in non-extracted teeth [11, 12]; however, differentiating a true MMC from an isthmus may not be possible [11], which may account for a higher incidence of MMC in the study of Azim et al. (46%) [11] compared with our study (4.15%).

The voxel size is crucial for detecting root canal system anatomy in CBCT studies. Mirmohammadi et al. has shown that a voxel size of 125 μm has an accuracy of 96% for detecting the second MB canal in maxillary molars [59]. Zhang et al. came to the same conclusion for choosing a voxel size of 125 μm when detecting root canal anatomy of mandibular premolars [60]. Vizzotto et al. reported that a CBCT voxel size of 200 μm had a higher sensibility than larger voxel sizes to detect the second MB canal [55]. Therefore, voxel sizes of more than 200 μm might miss some root canal anatomy. Thus, we excluded the studies that had advocated voxel sizes of more than 200 μm from the present study (Supplementary Figure S1). However, the analysis of the effect of different voxel sizes <200 μm on MMC detection has shown no significant differences [16]. In the study of Hatipoglu et al., the prevalence of MMC was equal (7%) for either voxel sizes ≤150 μm or 150–200 μm [16].

Field of view (FOV) is an important criterion in CBCT imaging. In endodontics, particularly for detecting root canals, a limited FOV CBCT is preferred over medium or large FOV CBCT [61]. This preference is due to the lower radiation dose to the patient, higher spatial resolution, and fewer volumes to interpret. As the FOV size increases, the spatial resolution and image quality decrease, resulting in a higher probability of missing a canal [61]. It should be noted that in morphological studies, CBCT images were not prescribed for endodontic reasons but for general aims such as surgical interventions or evaluation of the surrounding anatomies. Therefore, a medium to high field of view is commonly used, which can lead to underestimation of root canals, such as MMCs. The larger field of view is an inevitable limitation of studies included in the present meta-analysis.

Based on the recommendation of the joint position statement of the American Association of Endodontists and the American Academy of Oral and Maxillofacial Radiology, a limited FOV CBCT should be considered the imaging modality of choice for initial treatment of teeth with the potential for extra canals and suspected complex morphology [61]. As mentioned before, along with a limited FOV, voxel sizes ≤200 μm should be set for endodontic evaluations [55], such as detecting MMCs.

DOM is another essential aid in nonsurgical and surgical endodontics for locating additional canals [62]. In particular, performing a standardized troughing under high magnification between MB and ML canals is suggested to search for a MMC [11]. To manage the MMC canal during apical surgery of mandibular molars, Pomeranz et al. suggested that after resecting the apex and retro-preparation of the MB and ML canals, to deeply connect the canals to satisfactorily debride and to allow for good retention and sealing of the retrograde filling material [6]. Although the prevalence of MMC was rare in all geographic populations included in this study, the authors suggest always looking for MMC when doing an endodontic treatment on mandibular molars.

A limitation of the present study is that most of the included studies did not include C-shaped and single-rooted teeth in their sample size for estimating the prevalence of MMC; therefore, in only one study [34], to calculate the total number of included teeth, we excluded C-shaped and single-rooted teeth from the original sample size in that study. This procedure was performed to match and adjust their methodology to the other included studies. Although C-shaped and single-rooted molars are rare, excluding such teeth from the study may result in an over-representation of the remaining teeth, leading to an overestimation of the prevalence of MMC. Based on the topic of the present review, only cross-sectional studies could be inserted that, in the hierarchy of evidence, were considered as low level of evidence. However, 94.1% of the included studies had low bias levels.

When interpreting the results of the present study, it is important to consider the limited number of studies available in certain regions. For example, only a single study was conducted on the vast continent of North America [10]. Similarly, countries like Libya and Germany, which are larger than their neighbors, had only one study that met the inclusion criteria for our meta-analysis [16]. The scarcity of studies in these regions may explain the higher prevalence of MMCs compared to their neighboring countries. Among the included studies, only a few countries had two or more studies on the prevalence of MMCs. These countries included China, India, Iran, Pakistan, Yemen, Saudi Arabia, Portugal, and Brazil. Furthermore, there are still many countries for which we were unable to find any data regarding the prevalence of MMCs. Therefore, caution should be employed when generalizing the results of this meta-analysis to each region. It seems that further studies are needed on the prevalence of MMCs in different regions of the world.

For future studies, it is suggested that 1) studies must define their precise definition of a MMC versus isthmus so that it is easier to interpret the data, 2) perform studies on patients over multiple decades of age to determine how age may affect the detectability of MMC, and 3) consider a voxel size equal to or lower than 200 μm.

## Conclusion

The prevalence of MMC varies among regions. The MMC prevalence in mandibular 1^st^ molars is 4.15% globally. The mandibular 2^nd^ molar rarely has the MMC (1.2%). Understanding the incidence of MMC can guide clinicians as to whether troughing under high magnification or further investigation with CBCT is indicated when performing endodontic treatment of mandibular molars.

### Supplementary information

ESM 1 (JPG 13.9 kb)

ESM 2 (DOCX 51 kb)

ESM 3 (JPG 11.9 mb)

ESM 4 (JPEG 737 kb)

## Data Availability

No datasets were generated or analysed during the current study.
