# Supplementary material for: A systematic review and meta-analysis of the prevalence and global distribution of middle mesial canals in mandibular molars identified by CBCT
Source: Clin Oral Investig. 2024 May 14;28(6):310. doi: 10.1007/s00784-024-05660-z (PMC11093850; doi:10.1007/s00784-024-05660-z)
Supplement: Supplementary file 1 — (JPG 13.9 kb) [file 784_2024_5660_MOESM1_ESM.docx]

**Supplementary Table S1.** Search strategy with appropriate keywords and MeSH terms.

| **Level** | **Keywords** |
| --- | --- |
| Primary keywords combination for retrieving mandibular studies | (lower) OR (mandibular) |
| Primary keywords combination for retrieving teeth studies | (tooth) OR (teeth) OR (molar)) OR (molars) |
| Primary keywords combination for retrieving canal studies | (root) OR (roots)) OR (canal)) OR (canals) |
| Primary keywords combination for retrieving mid-mesial studies | (anatomy) OR (morphology) OR (configuration) OR (midmesial) OR (mid-mesial) OR (middle mesial) OR (middle-mesial) OR (variation) |
| Primary keywords combination for retrieving CBCT studies | (cbct) OR (cone-beam computed tomography)) OR (micro-computed tomography) OR (microcomputed tomography) OR (micro ct) OR (micro-ct)) OR (x-ray microtomography) |
| Adding relevant MeSH terms and field operators for PubMed retrieving mandibular studies | ("lower"[All Fields] OR "lowered"[All Fields] OR "lowering"[All Fields] OR "lowerings"[All Fields] OR "lowers"[All Fields] OR ("mandible"[MeSH Terms] OR "mandible"[All Fields] OR "mandibular"[All Fields] OR "mandibulars"[All Fields]) |
| Adding relevant MeSH terms and field operators for PubMed retrieving teeth studies | ("teeth s"[All Fields] OR "teeths"[All Fields] OR "tooth"[MeSH Terms] OR "tooth"[All Fields] OR "teeth"[All Fields] OR "tooth s"[All Fields] OR "tooths"[All Fields] OR ("teeth s"[All Fields] OR "teeths"[All Fields] OR "tooth"[MeSH Terms] OR "tooth"[All Fields] OR "teeth"[All Fields] OR "tooth s"[All Fields] OR "tooths"[All Fields]) OR ("molar"[MeSH Terms] OR "molar"[All Fields] OR "molars"[All Fields] OR "molar s"[All Fields]) OR ("molar"[MeSH Terms] OR "molar"[All Fields] OR "molars"[All Fields] OR "molar s"[All Fields])) |
| Adding relevant MeSH terms and field operators for PubMed retrieving canal studies | ("canal s"[All Fields] OR "canaled"[All Fields] OR "canals"[All Fields] OR "dental pulp cavity"[MeSH Terms] OR ("dental"[All Fields] AND "pulp"[All Fields] AND "cavity"[All Fields]) OR "dental pulp cavity"[All Fields] OR "canal"[All Fields]) OR ("canal s"[All Fields] OR "canaled"[All Fields] OR "canals"[All Fields] OR "dental pulp cavity"[MeSH Terms] OR ("dental"[All Fields] AND "pulp"[All Fields] AND "cavity"[All Fields]) OR "dental pulp cavity"[All Fields] OR "canal"[All Fields])) AND ("anatomy and histology"[MeSH Subheading] OR ("anatomy"[All Fields] AND "histology"[All Fields]) OR "anatomy and histology"[All Fields] OR "anatomy"[All Fields] OR "anatomy"[MeSH Terms] OR "anatomies"[All Fields] OR ("anatomy and histology"[MeSH Subheading] OR ("anatomy"[All Fields] AND "histology"[All Fields]) OR "anatomy and histology"[All Fields] OR "morphology"[All Fields] OR "morphologies"[All Fields]) OR ("configurability"[All Fields] OR "configurable"[All Fields] OR "configuration"[All Fields] OR "configurational"[All Fields] OR "configurations"[All Fields] OR "configure"[All Fields] OR "configured"[All Fields] OR "configures"[All Fields] OR "configuring"[All Fields]) OR "midmesial"[All Fields] OR "mid-mesial"[All Fields] OR (("middle"[All Fields] OR "middles"[All Fields]) |
| Adding relevant MeSH terms and field operators for PubMed retrieving middle mesial canal studies | ("mesial"[All Fields] OR "mesialization"[All Fields] OR "mesialized"[All Fields] OR "mesially"[All Fields])) OR "middle-mesial"[All Fields] OR ("variation"[All Fields] OR "variations"[All Fields]) |
| Adding relevant MeSH terms and field operators for PubMed retrieving CBCT studies | ("cbct"[All Fields] OR ("cone beam computed tomography"[MeSH Terms] OR ("cone beam"[All Fields] AND "computed"[All Fields] AND "tomography"[All Fields]) OR "cone beam computed tomography"[All Fields] OR ("cone"[All Fields] AND "beam"[All Fields] AND "computed"[All Fields] AND "tomography"[All Fields]) OR "cone beam computed tomography"[All Fields]) OR ("micro-computed"[All Fields] AND ("tomographie"[All Fields] OR "tomography"[MeSH Terms] OR "tomography"[All Fields] OR "tomographies"[All Fields] OR "tomography s"[All Fields] OR "tomography, x ray computed"[MeSH Terms] OR ("tomography"[All Fields] AND "x ray"[All Fields] AND "computed"[All Fields]) OR "x-ray computed tomography"[All Fields] OR "tomographys"[All Fields])) OR ("x ray microtomography"[MeSH Terms] OR ("x ray"[All Fields] AND "microtomography"[All Fields]) OR "x ray microtomography"[All Fields] OR ("microcomputed"[All Fields] AND "tomography"[All Fields]) OR "microcomputed tomography"[All Fields]) OR ("x ray microtomography"[MeSH Terms] OR ("x ray"[All Fields] AND "microtomography"[All Fields]) OR "x ray microtomography"[All Fields] OR ("micro"[All Fields] AND "ct"[All Fields]) OR "micro ct"[All Fields]) OR ("x ray microtomography"[MeSH Terms] OR ("x ray"[All Fields] AND "microtomography"[All Fields]) OR "x ray microtomography"[All Fields] OR ("micro"[All Fields] AND "ct"[All Fields]) OR "micro ct"[All Fields]) OR ("x ray microtomography"[MeSH Terms] OR ("x ray"[All Fields] AND "microtomography"[All Fields]) OR "x ray microtomography"[All Fields] OR "x ray microtomography"[All Fields])) |

Each step combined to the relevant step with AND Boolean operator.
