# Supplementary material for: A systematic review and meta-analysis of the prevalence and global distribution of middle mesial canals in mandibular molars identified by CBCT
Source: Clin Oral Investig. 2024 May 14;28(6):310. doi: 10.1007/s00784-024-05660-z (PMC11093850; doi:10.1007/s00784-024-05660-z)
Supplement: Supplementary file 2 — (DOCX 51 kb) [file 784_2024_5660_MOESM2_ESM.docx]

**Supplementary Table S2.** Excluded articles and reasons for exclusion (n=54)

| **First author, year** | **Reasons for exclusion** | **Number of articles** |
| --- | --- | --- |
| Akbarzadeh, 2017 [63]  Aldosimani, 2021 [64]  Choupani, 2018 [65]  Deng, 2018 [66]  Kenawi, 2022 [67]  Celikten, 2016 [68]  Madani, 2017 [69]  Nur, 2014 [70]  Pan, 2019 [26]  Srivastava, 2018 [27]  Talabani, 2022 [71]  Tredoux,2021 [72]  Yang, 2022 [73]  Alhujhuj, 2022 [74]  Fenelon, 2022 [75]  Erkan, 2023 [76]  Buchanan, 2023 [77] | Voxel size more than 200 µm for all of or a number of study samples | 17 |
| Al Shehadat, 2019 [78]  Demirbuga, 2013 [79]  Felsypremila, 2015 [80]  Huang, 2010 [81]  Kashyap, 2017 [82]  Lili, 2017 [83]  Pham, 2019 [84]  Mantovani, 2022 [85]  Guo, 2022 [86]  Rasidi, 2020 [87] | No report for voxel size or details of the CBCT device | 10 |
| Estrela, 2015 [88]  Beshkenadzeh, 2015 [89]  Mashyakhy, 2019 [90]  Mashyakhy, 2019 [91]  Mashyakhy, 2021 [92]  Mashyakhy, 2021 [93]  Monsarrat, 2016 [94] Razumova, 2018 [95]  Shigefuji, 2022 [96]  Almansour, 2022 [97] | Lack of details about the number of canals or canal configurations in the mesial root of mandibular molars | 10 |
| Mishra, 2020 [98]  Abarca, 2020 [99] | Did not report the prevalence of MMC separately in mandibular 1^st^ and 2^nd^ molars | 2 |
| Gambarini, 2018 [100]  Plotino, 2013 [101] | No inclusion/exclusion criteria | 2 |
| Martins, 2017 [102]  Martins, 2018 [103]  Martins, 2018 [104] | Used the same or in a common dataset as [46] that was already included | 3 |
| Mukhaimer, 2014 [105] | In vitro study | 1 |
| Honap, 2020 [106]  Weinberg, 2020 [107] | 1) In vitro studies  2) Did not report the prevalence of MMC separately in 1^st^ and 2^nd^ molars. | 2 |
| Kuzekanani, 2020 [108]  Barros-Costa, 2022 [109] | Included root-filled teeth | 2 |
| Perlea, 2019 [110] | 1) Included root-filled teeth  2) No inclusion or exclusion criteria | 1 |
| Nazeer, 2019 [111] | No clarity about Vertucci type VIII and additional modifications of Vertucci’s canal types | 1 |
| Torres, 2015 [112] | Did not report the total number of teeth | 1 |
| Przesmycka, 2019 [113]  Przesmycka, 2020 [114] | Anthropologic studies | 2 |
| *MMC: middle mesial canal* | | |

References:

63. Akbarzadeh N, Aminoshariae A, Khalighinejad N, Palomo JM, Syed A, Kulild JC, Sadeghi G, Mickel A (2017) The association between the anatomic landmarks of the pulp chamber floor and the prevalence of middle mesial canals in mandibular first molars: an in vivo analysis. J Endod 43:1797–1801.

https://doi.org/10.1016/j.joen.2017.07.003

64. Aldosimani MA, Althumairy RI, Alzahrani A, Aljarbou FA, Alkatheeri MS, AlGhizzi MA, Abughosh TK (2021) The mid-mesial canal prevalencein mandibular molars of a Saudi population: a cone-beam computed tomography study. Saudi Dent J 33:581–586

65. Choupani Dastgerdi A, Navabi M, Hafezi L, Khalilak Z, Rakhshan V (2018) Anatomy of permanent mandibular first molars in a selected Iranianpopulation using cone-beam computed tomography. Iran Endod J 13:251–256.

https://doi.org/10.22037/iej.v13i2.19035

66. Deng PU, Halim MS, Masudi SM, Al-Shehadat S, Ahmad B (2018) Cone-beam computed tomography analysis on root and canal morphology ofmandibular first permanent molar among multiracial population in East Coast Malaysian population. Eur J Dent 12:410–416.

https://doi.org/10.4103/ejd.ejd_82_18

67. Kenawi L, Althobaiti R, Filimban D, Alotaiby S, Alharbi M, Kassar W (2022, 2022) Radiographic investigation of root canal morphology of permanent mandibular molars in Makkah population (Saudi Arabia) using cone-beam computed tomography. Int J Dent 2022

68. Celikten B, Tufenkci P, Aksoy U, Kalender A, Kermeoglu F, Dabaj P, Orhan K (2016) Cone beam CT evaluation of mandibular molar root canalmorphology in a Turkish Cypriot population. Clin Oral Investig 20:2221–2226.

https://doi.org/10.1007/s00784-016-1742-2

69. Madani Z, Mehraban N, Moudi E, Bijani A (2017) Root and canal morphology of mandibular molars in a selected Iranian population using cone-beam computed tomography. Iran Endod J 12:143–148.

https://doi.org/10.22037/iej.2017.29

70. Nur BG, Ok E, Altunsoy M, Aglarci OS, Colak M, Gungor E (2014) Evaluation of the root and canal morphology of mandibular permanent molars in a south-eastern Turkish population using cone-beam computed tomography. Eur J Dent 8:154–159.

https://doi.org/10.4103/1305-7456.130584

71. Talabani R, Abdalrahman K, Abdul R, Babarasul D, Hilmi Kazzaz S (2022) Evaluation of radix entomolaris and middle mesial canal in mandibular permanent first molars in an Iraqi subpopulation using cone-beam computed tomography. BioMed Res Int 2022

72. Tredoux S, Warren N, Buchanan G (2021) Root and canal configurations of mandibular first molars in a South African subpopulation. J Oral Sci63:252–256

73. Yang L, Han J, Wang Q, Wang Z, Yu X, Du Y (2022) Variations of root and canal morphology of mandibular second molars in Chinese individuals: a cone-beam computed tomography study. BMC Oral Health 22:1–12

74. Alhujhuj RR, Jouhar R, Ahmed MA, Almujhim AA, Albutayh MT, Adanir N (2022) Evaluation of root canal configuration of maxillary and mandibular first molar by CBCT: a retrospective cross-sectional study. Diagnostics 12:2121

75. Fenelon T, Parashos P (2022) Prevalence and morphology of C-shaped and non-C-shaped root canal systems in mandibular second molars. AustEndod J 67:S65–S75

76. Erkan E, Olcay K, Eyüboğlu TF, Şener E, Gündoğar M (2023) Assessment of the canal anatomy of the mandibular molars in a group of Turkish patients: a cone-beam computed tomography study. Van Sağ Bil Derg 16:44–52

77. Buchanan GD, Gamieldien MY, Fabris-Rotelli I, van Schoor A, Uys A (2023) Investigation of mandibular second molar root and canal morphology in a Black South African population using cone-beam computed tomography and two classification systems. J Oral Sci 65:226–231

78. Al Shehadat S, Waheb S, Al Bayatti SW, Kheder W, Khalaf K, Murray CA (2019) Cone beam computed tomography analysis of root and root canal morphology of first permanent lower molars in a middle east subpopulation. J Int Soc Prev Community Dent 9:458–463.

https://doi.org/10.4103/jispcd.JISPCD_41_19

79. Demirbuga S, Sekerci AE, Dinçer AN, Cayabatmaz M, Zorba YO (2013) Use of cone-beam computed tomography to evaluate root and canal morphology of mandibular first and second molars in Turkish individuals. Med Oral Patol Oral Cir Bucal 18:e737–e744.

https://doi.org/10.4317/medoral.18473

80. Felsypremila G, Vinothkumar TS, Kandaswamy D (2015) Anatomic symmetry of root and root canal morphology of posterior teeth in Indian subpopulation using cone beam computed tomography: a retrospective study. Eur J Dent 9:500–507.

https://doi.org/10.4103/1305-7456.172623

81. Huang CC, Chang YC, Chuang MC, Lai TM, Lai JY, Lee BS, Lin CP (2010) Evaluation of root and canal systems of mandibular first molars in Taiwanese individuals using cone-beam computed tomography. J Formos Med Assoc 109:303–308.

https://doi.org/10.1016/s0929-6646(10)60056-3

82. Kashyap R, Beedubail S, Kini R, Rao P (2017) Assessment of the number of root canals in the maxillary and mandibular molars: a radiographic study using cone beam computed tomography. J Conserv Dent 20:288–291.

https://doi.org/10.4103/jcd.Jcd_73_17

83. Lili Y, Yan Z, Shijun Z, Shuai Z, Na W, Jie X, Shue H, Zhiyuan X (2017) Clinical application of cone beam computed tomography combined with micro-ultrasound technique in treating three mesial canals in mandibular first molars. West China J Stomatol 35:384–388

84. Pham KV, Le AHL (2019) Evaluation of roots and canal systems of mandibular first molars in a Vietnamese subpopulation using cone-beam computed tomography. J Int Soc Prev Community Dent 9:356–362.

https://doi.org/10.4103/jispcd.JISPCD_52_19

85. Mantovani VO, Gabriel AES, Silva RG, Savioli RN, Sousa-Neto MD, Cruz-Filho AM (2022) Analysis of the mandibular molars root canals morphology. Study by computed tomography. Braz Dent J 33:1–8

86. Guo Q, Wang Q, Yang Y, Guo D (2023) Root and root canal morphology of mandibular second permanent molars in the Gansu province population: a CBCT study. Aust Endod J 49:27–32

87. Rasidi MQZBM, Ramakrishnan M (2020) Root canal morphology of mandibular second molars using CBCT. Indian J Forensic Med Toxicol 14:162–167

88. Estrela C, Bueno MR, Couto GS, Rabelo LE, Alencar AH, Silva RG, Pécora JD, Sousa-Neto MD (2015) Study of root canal anatomy in human permanent teeth in a subpopulation of Brazil's center region using cone-beam computed tomography - part 1. Braz Dent J 26:530–536.

https://doi.org/10.1590/0103-6440201302448

89. Beshkenadze E, Chipashvili N (2015) Anatomo-morphological feutures of the root canal system in Georgian population - cone-beam computed tomography study. Georgian Med News 247:7–14

90. Mashyakhy M, Chourasia H, Halboub E, Almashraqi A, Khubrani Y, Gambarini G (2019) Anatomical variations and bilateral symmetry of rootsand root canal system of mandibular first permanent molars in Saudi Arabian population utilizing cone- beam computed tomography. Saudi DentJ 31:481–486.

https://doi.org/10.1016/j.sdentj.2019.04.001

91. Mashyakhy M, Gambarini G (2019) Root and root canal morphology differences between genders: a comprehensive in-vivo CBCT study in a Saudi population. Acta Stomatol Croat 53:213–246.

https://doi.org/10.15644/asc53/3/5

92. Mashyakhy M, Jabali A, Alabsi FS, AbuMelha A, Alkahtany M, Bhandi S (2021) Anatomical evaluation of mandibular molars in a Saudi population: an in vivo cone-beam computed tomography study. Int J Dent 2021:5594464.

https://doi.org/10.1155/2021/5594464

93. Mashyakhy M, Vinothkumar T, Arthisri A, Renugalakshmi A, Alamir A, Alkahtany M, Juraybi A (2021) Ethnical anatomical differences in mandibular first permanent molars between Indian and Saudi Arabian subpopulations: a retrospective cross-sectional study. J Contemp Dent Pract 22:484–490

94. Monsarrat P, Arcaute B, Peters OA, Maury E, Telmon N, Georgelin-Gurgel M, Maret D (2016) Interrelationships in the variability of root canal anatomy among the permanent teeth: a full-mouth approach by cone-beam CT. PLoS One 11:e0165329.

https://doi.org/10.1371/journal.pone.0165329

95. Razumova S, Brago A, Khaskhanova L, Howijieh A, Barakat H, Manvelyan A (2018) A cone-beam computed tomography scanning of the root canal system of permanent teeth among the Moscow population. Int J Dent 2018:2615746.

https://doi.org/10.1155/2018/2615746

96. Shigefuji R, Serikawa M, Usami A (2022) Observation of mandibular second molar roots and root canal morphology using dental cone-beam computed tomography. Anat Cell Biol 55:155–160.

https://doi.org/10.5115/acb.22.050

97. Almansour MI, Al-Zubaidi SM, Enizy AS, Madfa AA (2022) Comprehensive evaluation of root and root canal morphology of mandibular secondmolars in a Saudi subpopulation evaluated by cone-beam computed tomography. BMC Oral Health 22:1–9

98. Mishra P, Sahu Y, Malik R, Pal A, Sharma S, Tamrakar R (2020) Incidence of middle mesial canals based on distance between mesial canal orifices in mandibular molars: cone-beam computed tomographic analysis. J Adv Med Dent Sci Res 8:4–7

99. Abarca J, Duran M, Parra D, Steinfort K, Zaror C, Monardes H (2020) Root morphology of mandibular molars: a cone-beam computed tomography study. Folia Morphol 79:327–332

100. Gambarini G, Piasecki L, Ropini P, Miccoli G, Nardo DD, Testarelli L (2018) Cone-beam computed tomographic analysis on root and canal morphology of mandibular first permanent molar among multiracial population in Western European population. Eur J Dent 12:434–438.

https://doi.org/10.4103/ejd.ejd_116_18

101. Plotino G, Tocci L, Grande NM, Testarelli L, Messineo D, Ciotti M, Glassman G, D'Ambrosio F, Gambarini G (2013) Symmetry of root and root canal morphology of maxillary and mandibular molars in a white population: a cone-beam computed tomography study in vivo. J Endod 39:1545–1548.

https://doi.org/10.1016/j.joen.2013.09.012

102. Martins J, Marques D, Mata A, Caramês J (2017) Root and root canal morphology of the permanent dentition in a Caucasian population: a cone-beam computed tomography study. Int Endod J 50:1013–1026.

https://doi.org/10.1111/iej.12724

103. Martins J, Marques D, Francisco H, Caramês J (2018) Gender influence on the number of roots and root canal system configuration in human permanent teeth of a Portuguese subpopulation. Quintessence Int 49:103–111.

https://doi.org/10.3290/j.qi.a39508

104. Martins J, Ordinola-Zapata R, Marques D, Francisco H, Caramês J (2018) Differences in root canal system configuration in human permanent teeth within different age groups. Int Endod J 51:931–941.

https://doi.org/10.1111/iej.12896

105. Mukhaimer RH (2014) Evaluation of root canal configuration of mandibular first molars in a Palestinian population by using cone-beam computed tomography: an ex vivo study. Int Sch Res Notices 2014:583621.

https://doi.org/10.1155/2014/583621

106. Honap MN, Devadiga D, Hegde MN (2020) To assess the occurrence of middle mesial canal using cone-beam computed tomography and dentaloperating microscope: an in vitro study. J Conserv Dent 23:51–56.

https://doi.org/10.4103/jcd.Jcd_462_19

107. Weinberg EM, Pereda AE, Khurana S, Lotlikar PP, Falcon C, Hirschberg C (2020) Incidence of middle mesial canals based on distance betweenmesial canal orifices in mandibular molars: a clinical and cone-beam computed tomographic analysis. J Endod 46:40–43.

https://doi.org/10.1016/j.joen.2019.10.017

108. Kuzekanani M, Walsh L, Amiri M (2020) Prevalence and distribution of the middle mesial canal in mandibular first molar teeth of the Kerman population: a CBCT study. Int J Dent 2020:8851984.

https://doi.org/10.1155/2020/8851984

109. Barros-Costa M, Diniz MF, Costa FF, Freitas DQ (2022) Middle mesial root canals in mandibular molars: prevalence and correlation to anatomical aspects based on CBCT imaging. Dentomaxillofac Radiol:20220156.

https://doi.org/10.1259/dmfr.20220156

110. Perlea P, Temelcea AN, Nistor CC, Gheorghiu IM, Iliescu AA (2019) Incidence of the middle mesial canals in mandibular permanent molars ina Romanian population by cone-beam computed tomography. Rom J Morphol Embryol 60:1285–1290

111. Nazeer MR, Khan FR (2019) Evaluation of the root and canal morphology of mandibular first permanent molars in a sample of Pakistani population by cone-beam computed tomography. J Pak Med Assoc 69:1084–1089

112. Torres A, Jacobs R, Lambrechts P, Brizuela C, Cabrera C, Concha G, Pedemonte ME (2015) Characterization of mandibular molar root and canal morphology using cone beam computed tomography and its variability in Belgian and Chilean population samples. Imaging Sci Dent 45:95–101.

https://doi.org/10.5624/isd.2015.45.2.95

113. Przesmycka A, Tomczyk J, Pogorzelska A, Regulski P, Szopiński K (2019) Detection of root canals in historical population from Radom (Poland). Folia Morphol (Warsz) 78:853–861.

https://doi.org/10.5603/FM.a2019.0014

114. Przesmycka A, Jędrychowska-Dańska K, Masłowska A, Witas H, Regulski P, Tomczyk J (2020) Root and root canal diversity in human permanent maxillary first premolars and upper/lower first molars from a 14th-17th and 18th-19th century Radom population. Arch Oral Biol 110:104603.

https://doi.org/10.1016/j.archoralbio.2019.104603
