# Supplementary figures and images for: A systematic review and meta-analysis of the prevalence and global distribution of middle mesial canals in mandibular molars identified by CBCT
Source: Clin Oral Investig. 2024 May 14;28(6):310. doi: 10.1007/s00784-024-05660-z (PMC11093850; doi:10.1007/s00784-024-05660-z)

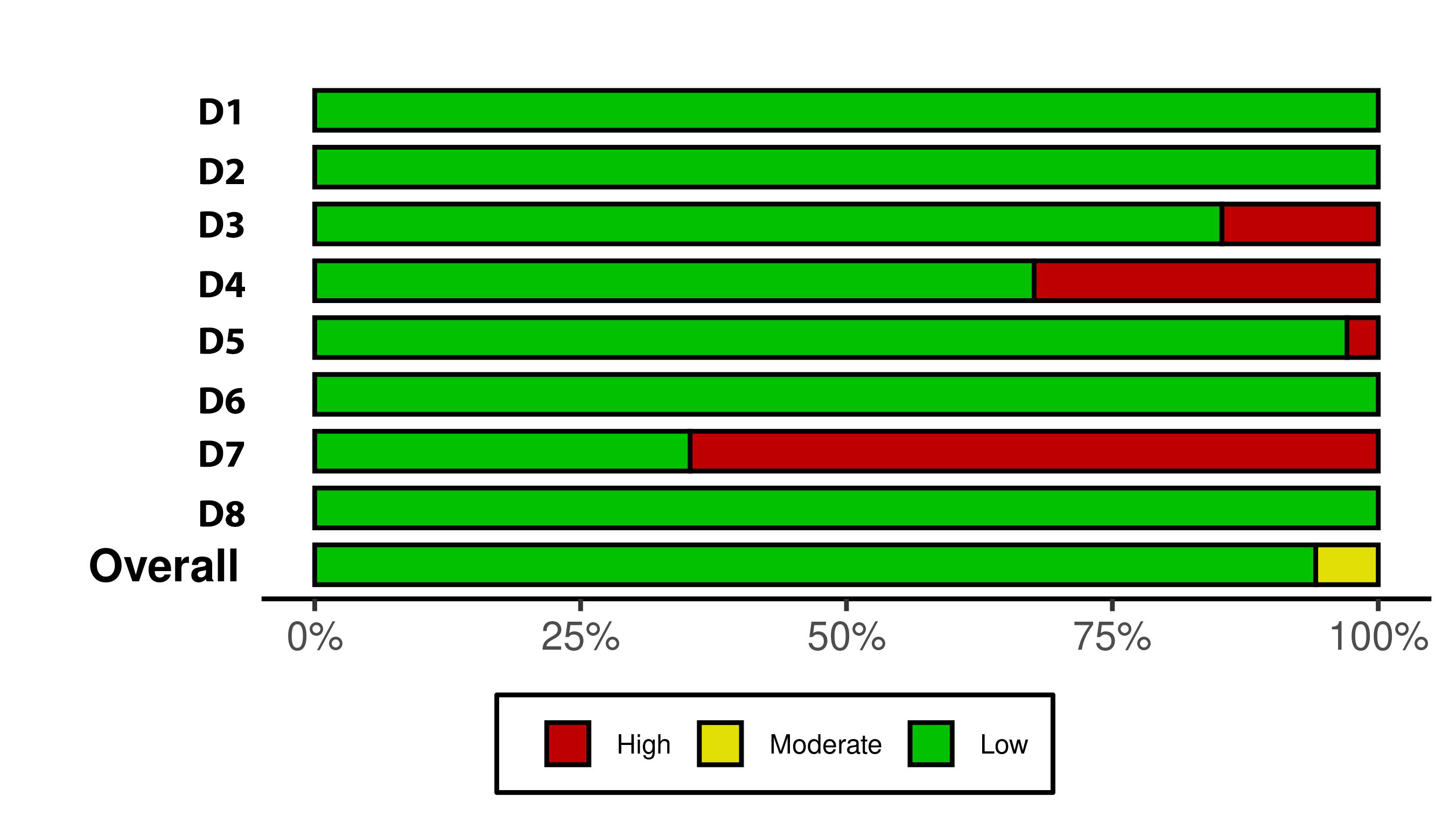

Supplement: Supplementary file 4 — (JPEG 737 kb) [file 784_2024_5660_MOESM4_ESM.jpeg]
